# Recent Treatment Advances and the Role of Nanotechnology, Combination Products, and Immunotherapy in Changing the Therapeutic Landscape of Acute Myeloid Leukemia

**DOI:** 10.1007/s11095-019-2654-z

**Published:** 2019-06-24

**Authors:** Kent T. J. Chen, Roger Gilabert-Oriol, Marcel B. Bally, Ada W. Y. Leung

**Affiliations:** 1 Department of Experimental Therapeutics, BC Cancer Research Centre, Vancouver, British Columbia Canada; 2 Department of Interdisciplinary Oncology, BC Cancer Research Centre, Vancouver, British Columbia Canada; 3 Department of Pathology and Laboratory Medicine, University of British Columbia, Vancouver, British Columbia Canada; 4 Cuprous Pharmaceuticals Inc., Vancouver, British Columbia Canada; 5 Department of Chemistry, University of British Columbia, Vancouver, British Columbia Canada

**Keywords:** acute myeloid leukemia, immunotherapy, liposomes, nanotechnology

## Abstract

Acute myeloid leukemia (AML) is the most common acute leukemia that is becoming more prevalent particularly in the older (65 years of age or older) population. For decades, “7 + 3” remission induction therapy with cytarabine and an anthracycline, followed by consolidation therapy, has been the standard of care treatment for AML. This stagnancy in AML treatment has resulted in less than ideal treatment outcomes for AML patients, especially for elderly patients and those with unfavourable profiles. Over the past two years, six new therapeutic agents have received regulatory approval, suggesting that a number of obstacles to treating AML have been addressed and the treatment landscape for AML is finally changing. This review outlines the challenges and obstacles in treating AML and highlights the advances in AML treatment made in recent years, including Vyxeos®, midostaurin, gemtuzumab ozogamicin, and venetoclax, with particular emphasis on combination treatment strategies. We also discuss the potential utility of new combination products such as one that we call “EnFlaM”, which comprises an encapsulated nanoformulation of flavopiridol and mitoxantrone. Finally, we provide a review on the immunotherapeutic landscape of AML, discussing yet another angle through which novel treatments can be designed to further improve treatment outcomes for AML patients.

## Preface

In the last two years, eight new agents have received regulatory approval for the treatment of acute myeloid leukemia (AML), representing a breakthrough given that management of AML has relied primarily upon standard 7 + 3 chemotherapy for over four decades. What have we learned from the past failures and recent successes? How can we combine the lessons learned to create novel therapeutics that will further improve treatment outcomes in AML patients with poor prognosis? Through reviewing the clinical problems of AML along with the unique features of investigational and recently approved treatments, we came to these conclusions: First, the fact that six of the eight new AML treatments have shown superior efficacy and/or have been approved in the combination setting highlights the importance of using multiple agents to target the heterogeneous nature of the disease. Second, optimizing the manner through which broad spectrum agents or any therapeutic agent is administered could have a profound effect on treatment outcomes as demonstrated by the approval of Vyxeos®. Third, improved understandings of the biology of AML have contributed to more effective treatments by enabling resistance mechanisms to be targeted upfront (e.g. the use of MCL-1 inhibitors in combination with potent agents that are known to give rise to MCL-1 overexpressing relapses). Fourth, emerging concepts such as various vaccination approaches, immunogenic cell death, and immunotherapy adjuvants that stimulate the presentation of multiple cancer-derived antigens could be promising for a disease that is normally considered poorly immunogenic. Altogether, we believe that the most effective regimens for AML patients with unfavourable profiles would involve multi-modal treatments comprising combinations of chemotherapy, targeted agents, or immune-based treatments: therapeutic agents that may be further enhanced by leveraging the advantages of nanoscale drug delivery systems.

## Background - Acute Myeloid Leukemia

**Fig. 1:**
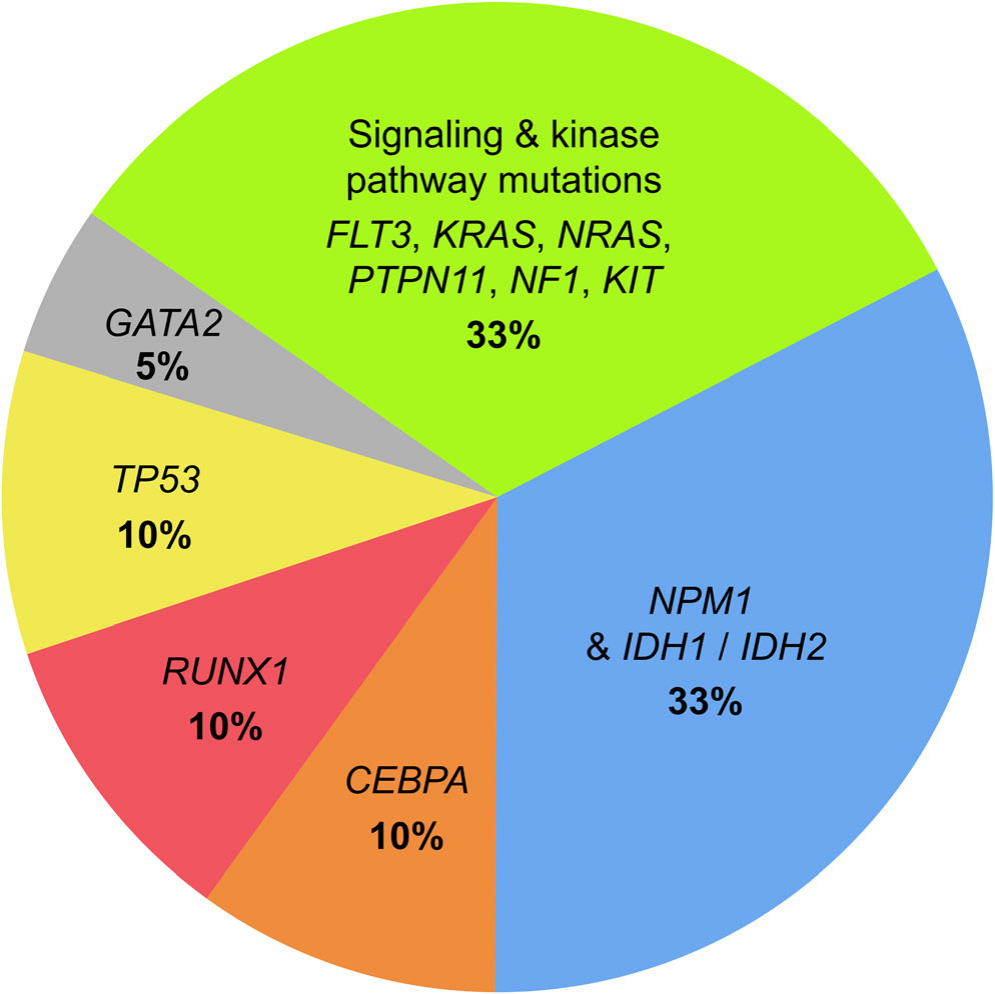
Most commonly found mutations in AML.

Acute myeloid leukemia (AML) is the most common myeloid leukemia and it is also becoming more prevalent amongst adults aged 65 years or older (1,2). AML can arise as a de novo malignancy in an otherwise healthy patient or as a secondary malignancy resulting from mutagenic events caused by leukemogenic agents such as alkylating chemotherapy drugs or radiation (3–5). AML was initially considered a monolithic disease that can be treated with a “one-for-all” chemotherapy drug (6). However, studies over the years revealed that AML is actually a genetically pleiomorphic disease, characterized by genetic heterogeneity at diagnosis (6,7). A summary of the mutational landscape of AML is presented in Fig. 1. Therefore, in order to capture this diversity to achieve better treatment outcomes, cytogenetic analysis has become a mainstay in the standard diagnosis of AML (6). Despite efforts to improve therapeutic outcomes and to better understand this disease, the standard of care for AML has remained largely unchanged for more than 40 years and it consists of two phases: remission induction therapy followed by consolidation therapy (2,8). Induction therapy consists of a “7 + 3” regimen which entails 7 days of continuous infusion of cytarabine in combination with 3 consecutive days of anthracycline treatment (typically daunorubicin) (8). Upon achieving initial remission from induction therapy, patients then undergo consolidation therapy to eradicate residual leukemia and this regimen typically includes high doses of cytarabine with or without hematopoietic stem cell transplantation (8,9). For most patients who are 60 years of age or younger, a complete remission is achievable with this initial cytotoxic induction therapy (7,10). However, despite the likelihood of achieving initial remission, AML remains challenging to treat, particularly when the patients are older or when the disease relapses or becomes refractory (10). However, the treatment landscape is changing due to (i) a better understanding of what is driving malignancy which points towards targeted therapeutics; (ii) the use of immunotherapy strategies; and (iii) the application of nanoformulation technology to prepare combination products. Here, the clinical challenges of AML, the recently developed therapeutic strategies, and emerging treatment designs are reviewed for the treatment of AML in the newly diagnosed as well as the relapsed or refractory (r/r) setting.

## Challenges of Treating AML

Management of AML in elderly patients is particularly challenging. Patients who are older than 65 years of age often present adverse cytogenetic risk profiles and these patients typically carry more comorbidities. In a retrospective analysis of 968 adult AML patients, the percentage of patients with unfavourable cytogenetics increased from 35% in patients younger than 56 years of age to 51% in those older than 75 (11). Furthermore, there was also an increase from 33% in younger patients to 57% in older patients when considering the percentage of patients that develop multidrug resistance (11). The increased incidence of unfavourable cytogenetics coupled with other comorbidities such as lower white blood cell counts make the older patients less tolerant of intense chemotherapy and hematopoietic stem cell transplantation (HSCT) (9,11,12). As a result, treatment outcome deteriorates markedly with age and the prognosis is particularly grim for older AML patients, with complete remission rates of less than 55% and a 5-year survival rate of less than 10% (9,13).

In addition to age, further challenges in treating AML arise when the disease relapses, and this applies to the majority of patients who achieved initial remission (10,14). The complexities and challenges of treating relapsed and refractory (r/r) AML often stem from the genetic heterogeneity presented at the time of diagnosis (15). Particularly, studies have demonstrated that AML features dynamic clonal evolution at relapse, with the appearance of new mutations that may contribute to the pathogenesis of r/r AML (16). In fact, this clonal evolution has been highlighted as the primary driver of chemotherapy resistance and the cause of treatment failure in r/r AML (16,17). One of the implicated genetic aberrations that has shown to be prominent in chemo-resistance in AML, contributing to relapses, is the elevated expression of proteins belonging to the Bcl-2 protein family, particularly Mcl-1 (18–20). Mcl-1 is a pro-survival, anti-apoptotic protein that primarily functions as an inhibitor of apoptosis effectors Bak and Bax (19,20). As such, elevated levels of Mcl-1 have been shown to delay apoptosis, leading to chemo-resistance and the ensuing relapse of the disease (18,20,21).

Over the past four decades, multiple clinical trials were conducted to examine alternative doses, schedule, and even new cytotoxic agents in an attempt to achieve therapeutic improvements (13,22). Unfortunately, AML patients still face the same therapeutic obstacles and relapse after complete remission (2,8,13). AML clearly presents an unmet medical need and new therapeutic strategies to improve treatment outcomes of AML, in particular r/r AML, are urgently required.

## Novel Therapeutic Strategies for AML

**Fig. 2:**
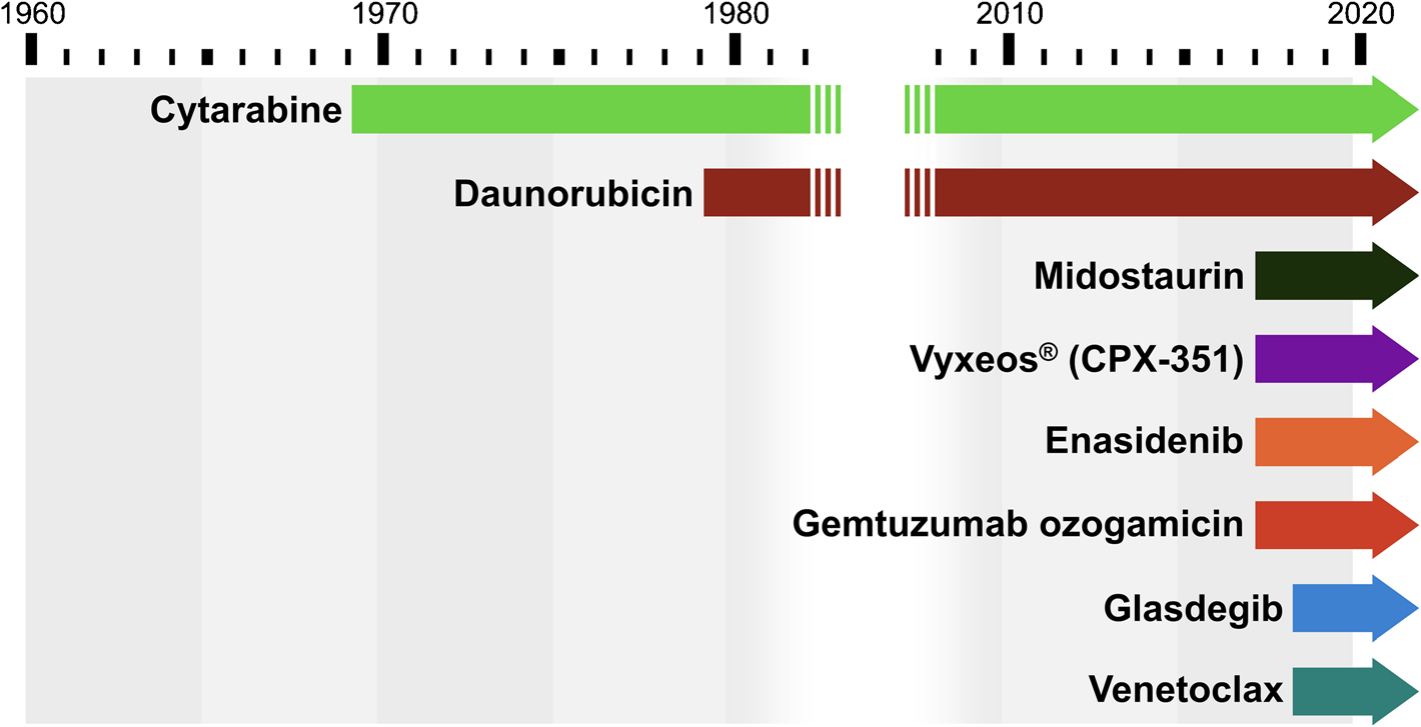
Progression of AML therapeutics over the years.

Given tumour heterogeneity and the ability of AML cells to activate a plethora of pro-survival signalling pathways, combination treatments that target multiple leukemogenic pathways is viewed as required to achieve improvements in overall survival in this patent population (23,24). Unfortunately, for decades, AML has been a therapeutic graveyard of failed drug programs designed to look for new treatment regimens. The vast majority of the therapeutic studies have typically come from tinkering with existing therapeutics such as increasing doses of daunorubicin and cytarabine for induction and consolidation therapy respectively or alternatively assessing daunorubicin replacements such as mitoxantrone or vosaroxin (25–28). Fortunately, there was a change in the therapeutic landscape for AML in 2017 and 2018 with novel therapeutic agents being approved by the Food and Drug Administration (FDA) for the treatment of AML (Fig. 2).

2017 saw the approval of four new therapeutic options by the FDA: Midostaurin (Novartis Pharmaceuticals), Enasidenib (Celgene and Agios Pharmaceuticals), Vyxeos® (also known as CPX 351; Celator/Jazz Pharmaceuticals), and Gemtuzumab ozogamicin (Pfizer) (29). Further progress was made in 2018 with the approval of Venetoclax (AbbVie and Genentech) and Daurismo™ (Pfizer). Ivosidenib (Agio Pharmaceuticals) and gilteritinib (Astellas Pharma US) were also approved as single-agent treatment for refractory and relapsed AML in 2018. However, as this review is focused on combination treatments, detailed discussions of ivosidenib and gilteritinib will not be pursued. Nevertheless, as we believe that combinations are capable of exhibiting efficacy far greater than single agent activities, new combinations utilizing new and improved single agents should be developed and investigated. The approval of these new therapeutics attempts to address the many challenges of AML treatment and has provided AML patients with alternatives where intensive chemotherapy is not an option. A summary of the therapeutic agents discussed below is presented in Table I.

**Table I:**
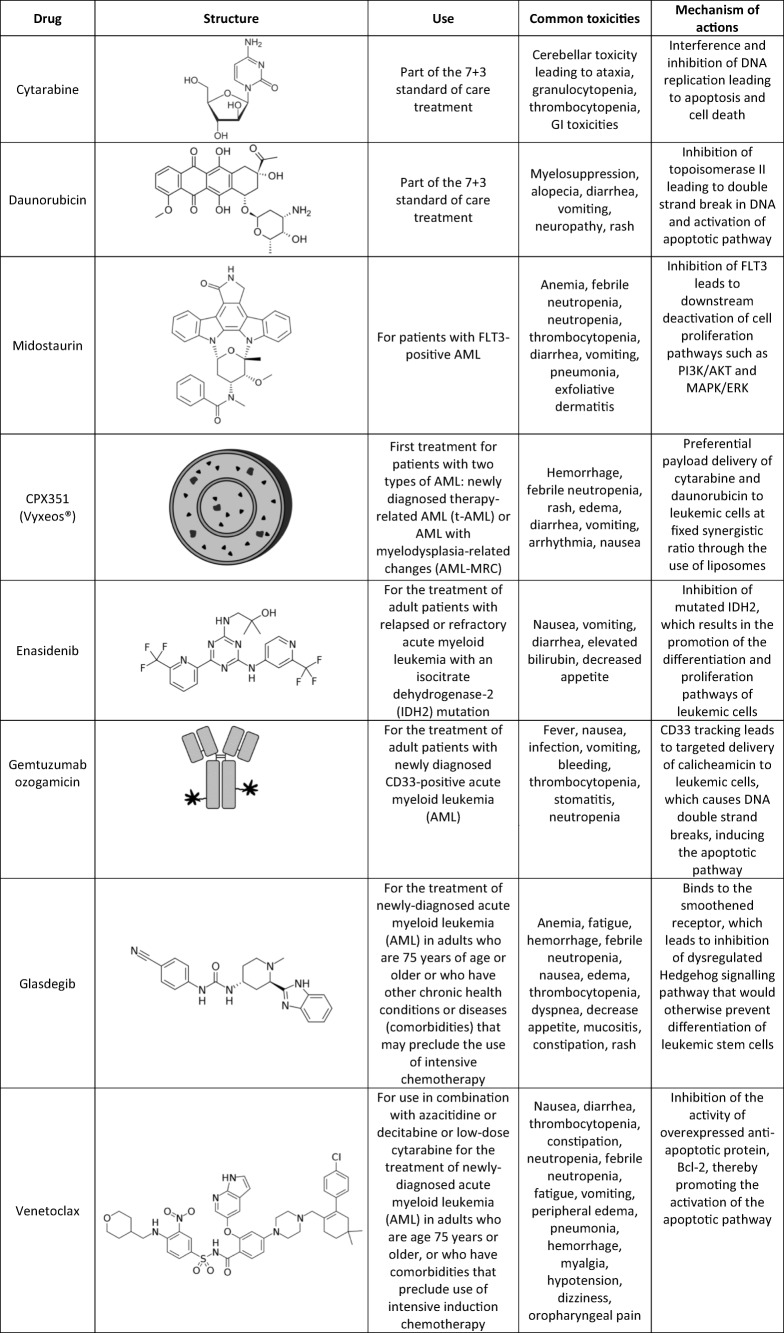
Therapeutic Landscape of AML

### Midostaurin

Some of the most common genetic aberrations found in AML are FLT3 mutations (30). Mutations of the tyrosine kinase FLT3 activate downstream pathways such as the PI3K and RAS/ERK pathways (31). FLT3 mutations can occur as internal tandem duplication (ITD) or can be found in the tyrosine kinase domain (TKD) (30,31). FLT3 mutations confer a negative prognosis in AML patients and have emerged as important targets for therapy (31). One of these promising inhibitors that specifically act against FLT3-TKD and FLT3-ITD mutants is midostaurin (30,32). As a single agent, midostaurin has limited utility in AML (30). However, when used in combination with standard of care “7 + 3” chemotherapy, midostaurin significantly prolongs overall survival as demonstrated in a phase 3 clinical trial with 717 patients, where FLT3-positive AML patients survived 49 months longer based on median survival and had a 5.2 month-improvement in event-free survival. Additionally, these patients had a 24.3% lower risk of death compared to just chemotherapy alone (33). In light of the positive data, FDA approved midostaurin on April 28th, 2017 to be used with the traditional standard of care (cycles of cytarabine and daunorubicin) for the treatment of adult AML patients with newly diagnosed FLT3-mutations (30). This marked the first new FDA approval for AML treatment in decades (32). Additionally, midostaurin is currently under investigation for use in combination with the aforementioned Vyxyeos® to treat patients with FLT3 mutations and preliminary findings revealed robust synergy when the two therapeutic agents were used concurrently (34).

### Enasidenib

Enasidenib is an isocitrate dehydrogenase 2 (IDH2) inhibitor (35). Isocitrate dehydrogenase mutations are observed in 15–20% of AML patients and cause the reduction of α-ketoglutarate to 2-hydroxyglutarate (36). 2-hydroxyglutarate inhibits the function of histone lysine demethylases, leading to hypermethylation of DNA and histones (36). This, in turn, exerts leukemogenic effects by blocking the differentiation of hematopoietic progenitor cells (36). Enasidenib was approved by the FDA on August 1st, 2017 as a monotherapy drug for the treatment of adult patients with r/r AML with IDH2 mutation. As a single agent, enasidenib is an effective option, with a complete remission (CR) rate of around 20% in AML patients having IDH2 mutations (35,37). However, enasidenib is significantly underutilized if it is only used as a monotherapy agent as enasidenib showed marked improvement in the CR rate when used in combination with standard induction chemotherapy (37). In a phase 1 clinical study with AML patients possessing IDH2 mutations, combining enasidenib with standard induction chemotherapy resulted in an improvement of CR rates to 67% and 58% in de novo and secondary AML respectively (37).

### CPX-351 (Vyxeos®)

CPX-351, or Vyxeos®, is a liposomal formulation of cytarabine and daunorubicin that delivers the two drugs at a fixed synergistic cytarabine-to-daunorubicin molar ratio of 5:1 (38,39). Compared to standard of care treatment, Vyxeos® demonstrated superior median overall survival (3.61 months longer), event-free survival (1.22 months longer), and remission rate (14.4% higher) without increasing treatment-related mortality and toxicities (38–40). Liposomes are well-established nanocarriers that are known to prolong circulation time in blood and this remarkable improvement in treatment outcome by Vyxeos® was attributed to the increased circulation lifetime of the cytotoxic agents as well as the administration and maintenance of a fixed synergistic drug combination ratio (40–42). Based on these positive findings, FDA approved Vyxeos® for use in the treatment of adults with newly diagnosed therapy-related AML on August 3, 2017 (43).

### Venetoclax

Venetoclax is an orally bioavailable Bcl-2 inhibitor (44). As discussed previously, overexpression of Bcl-2 implicates survival of AML cells and is often associated with treatment resistance (44). Inability to completely eradicate leukemic stem cells (LSC) is often believed to be an important cause of treatment failure and disease relapse in AML and high levels of Bcl-2 have recently been identified as a defining characteristic of LSC (45,46). As a Bcl-2 inhibitor, Venetoclax has shown promising activity when used in combination with hypomethylating agents such as azacitidine or decitabine or with low-dose cytarabine. These venetoclax combinations were granted accelerated approval from the FDA on November 21, 2018 to be used to treat adult AML patients who are aged 75 years or older or patients who have comorbidities that preclude them from being treated with induction chemotherapy (44,47). Complete remission rates were 37%, 54%, and 21% when venetoclax was used in combination with azacitidine, decitabine, and low-dose cytarabine respectively (44,47). Phase 3 studies (NCT02993523 and NCT03069352) are currently underway to serve as confirmatory trials to evaluate overall survival when venetoclax is used in combination with azacitidine or low-dose cytarabine. Additionally, venetoclax has shown synergy when used with MCL-1-inhibitors such as flavopiridol (alvocidib) and this combination is now being tested as a rational combination in phase I clinical trial (NCT03441555) (48).

### Glasdegib (Daurismo™)

The Hedgehog signalling pathway is critical to embryonic development by regulating differentiation and proliferation in a time-dependent fashion (49). Dysregulation of the Hedgehog pathway has been detected in a variety of hematopoietic malignancies, predominantly in leukemic stem cells (50,51). Leukemic stem cells are typically quiescent and are thus resistant to conventional chemotherapy treatments (51). Therefore, inhibitors of Hedgehog signalling may be ideal options to target leukemic stem cells. Glasdegib, or Daurismo™, is a small molecule inhibitor of the transmembrane protein Smoothened (SMO) (47). Translocation of SMO into the primary cilium of blood cells is a necessary step in the activation of the Hedgehog pathway (47,52). As a SMO inhibitor, glasdegib prevents this translocation and in turn inhibits the activation of downstream Hedgehog targets (47). Based on a randomized phase 2 trial (NCT01546038), the use of glasdegib with low dose cytarabine extended median overall survival by 3.4 months and improved complete response rates by 12.7% relative to using low-dose cytarabine alone (53). Given its promise, glasdegib was approved by the FDA on November 21st, 2018 to be used in combination with low-dose cytarabine to treat AML patients who are 75 years or older or those who have comorbidities that prohibit them from undergoing intensive induction chemotherapy.

### Gemtuzumab Ozogamicin

One of the most investigated AML-related target is CD33 (Siglec-3), an antigen that is highly expressed on blasts of 85–90% patients with AML as well as on normal myeloid cells (54). Expression of CD33 is highly variable with about half of AML patients expressing CD33 on >75% of leukemic blast cells (55). Although anti-CD33 monoclonal antibodies (mAbs) such as lintuzumab are tolerated in human clinical trials, they failed to provide meaningful clinical benefits (56,57). The only approved CD33-targeting agent is gemtuzumab ozogamicin (GO; Mylotarg™), a humanized CD33 mAb conjugated to a DNA damaging calicheamicin derivative (57). Gemtuzumab ozogamicin was granted FDA approval in 2000. However, based on a phase 3 trial (Southwest Oncology Group (SWOG); S0106 study; NCT00085709), the addition of GO to standard chemotherapy not only failed to improve treatment outcomes but was associated with statistically significant increase in treatment-related deaths relative to the standard of care (SOC) arm (55,58). This led to the voluntary withdrawal of Mylotarg™ by Pfizer in 2010. In September 2017, Mylotarg™ was re-approved by the FDA to be used in combination with daunorubicin and cytarabine for the treatment of newly diagnosed CD33+ AML patients and for r/r CD33+ adult and pediatric patients over the age of two. The approval was based on results from ALFA-0701 (NCT00927498), a multicenter phase III study of 271 newly diagnosed AML patients. Here, the addition of GO to standard chemotherapy extended median event-free survival by 8.2 months (no significant gain in overall survival) with a hazard ratio of 0.66 (57,59). In light of the success of Mylotarg™ in combination with standard of care chemotherapy, a phase I clinical study was initiated to investigate the potential of using Mylotarg™ with Vyxeos® to treat patients with r/r AML (NCT03672539).

The success of Mylotarg™ highlighted the potential of cancer immunotherapy in hematologic malignancies and to gain a better understanding of the immune aspects of AML, we review below the immunotherapy landscape for AML and discuss the challenges as well as opportunities of the immunotherapeutic strategies for the treatment of AML in the newly diagnosed as well as the relapsed or refractory (r/r) setting.

## Application of Immunotherapeutic Concepts for the Treatment of AML

**Fig. 3:**
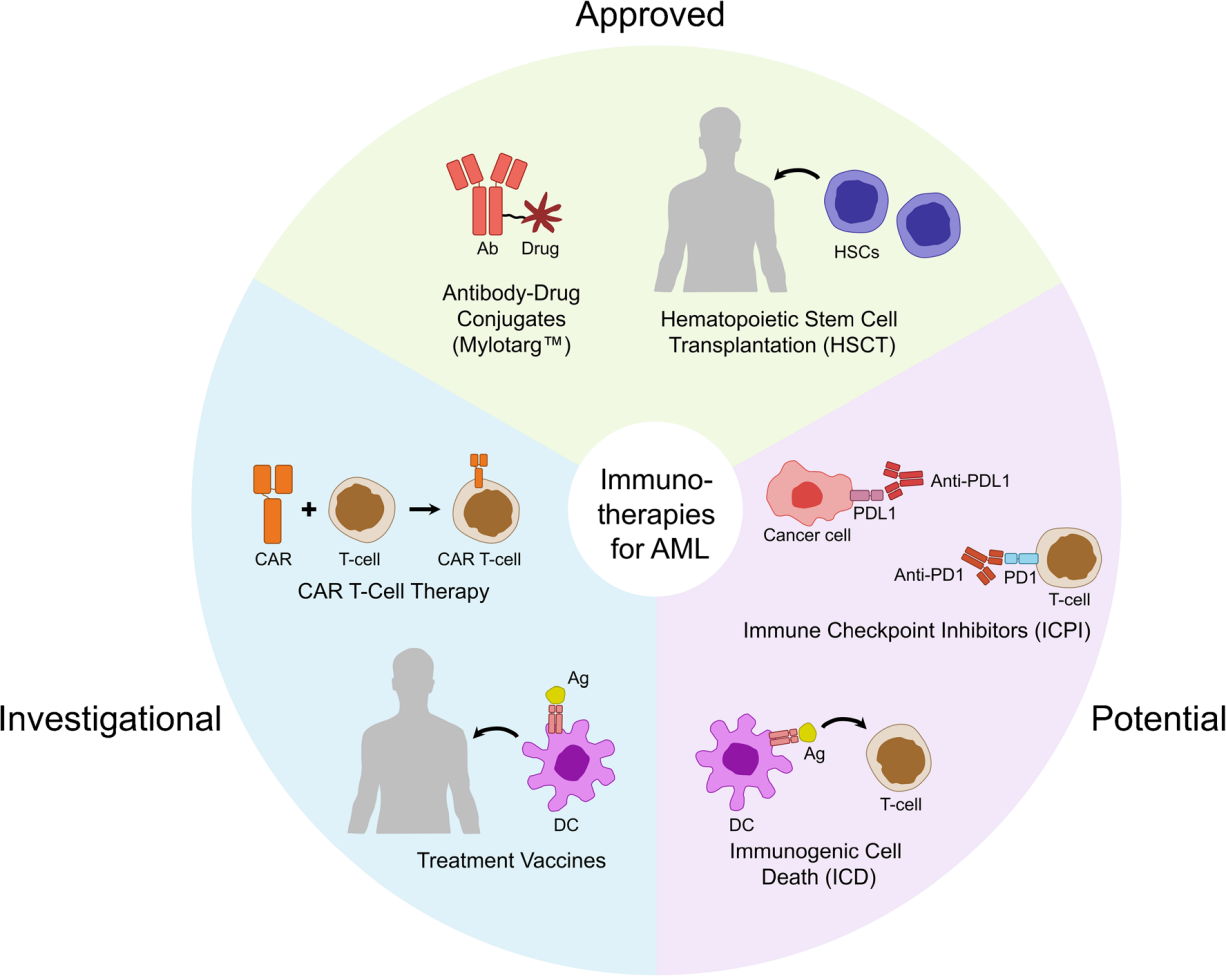
Immunotherapy strategies for AML treatment.

Advances in cancer immunotherapy over the past decade have undoubtedly revolutionized treatment strategies for both solid tumours and hematologic malignancies (60,61). Since 2011, six immune checkpoint inhibitors (ICPIs) have been approved by the FDA to treat various solid tumours including metastatic melanoma and advanced non-small cell lung cancer (NSCLC): illnesses that were considered untreatable at diagnosis until recent years (60). ICPIs have been associated with promising and durable responses extending survival, for the first time, for months or even years in patient populations that would otherwise succumb to their diseases within a year’s time. The use of genetically modified lymphocytes, specifically chimeric antigen receptor (CAR) T cells, on the other hand, has achieved great successes in the treatment of blood cancers, primarily B cell malignancies such as B cell acute lymphoblastic leukemia (ALL), chronic lymphoblastic leukemia (CLL), and non-Hodgkin’s lymphoma (57,61,62). The different immunotherapies that have been considered for treating AML are described in the following sections and are summarized in Fig. 3.

### Hematopoietic Stem Cell Transplantation (HSCT)

A small population of poor-risk AML patients may be given the option for allogeneic hematopoietic stem cell transplantation (HSCT) based on factors such as comorbidities, age, and availability of donors (63,64). HSCT is one of the oldest and most successful immunotherapeutic approaches in the treatment of AML, which involves ablation of the bone marrow using radiation or chemotherapy followed by re-implantation of stem cells from the host (autologous) or a donor (allogeneic) (57). Unfortunately, the majority of elderly patients are not eligible for HSCT, which is the last curative option for r/r patients (57,65). Even after HSCT, 40–60% patients develop graft-*versus*-host disease (GVHD) and approximately 20–50% patients invariably relapse and are left with very limited treatment options, underscoring the importance of developing novel immunotherapeutic approaches to improve this dire scenario (66,67). In recent years, efforts are being placed on optimising HSCT procedures, some of which include the use of reduced-intensity conditioning or myeloablative conditioning prior to HSCT (NCT02626715), infusion of donor natural killer cells (NCT03300492), and prophylactic use of IL-2 in combination with HSCT (NCT01517347), all of which are designed to lower the incidence of GVHD, reduce toxicities, extend the application of HSCT to elderly and frail patients, and to prevent relapse (68–70).

### Antibody-Drug Conjugates

Novel, non-transplant immunotherapeutic modalities are also being heavily explored for AML. In addition to the CD33-targeting agent, Mylotarg™, which was discussed above, Vadastuximab talirine (SGN-CD33A) is another antibody-drug conjugate (ADC) that has been tested in human clinical trials in recent years. While results from phase I study were promising, a phase III trial evaluating the combination of SGN-CD33A with the hypomethylating agents azacitidine or decitabine in newly diagnosed elderly AML patients was discontinued by Seattle Genetics in 2017 due to increased treatment-related mortality (71,72).

CD123 is another AML-target of interest particularly due to its more restricted expression on normal hematopoietic stem cells, suggesting the potential to reduce “on-target off-leukemia toxicities” (54,57,73,74). To date, talacotuzumab (mAb; J&J; NCT02472145) and SGN-CD123A (ADC; Seattle Genetics; NCT02848248 have been tested in human clinical trials, of which both were halted in development due to unfavourable risks/benefits profiles. Taken together, additional studies are warranted to validate the feasibility and utility of targeting CD123 in the treatment of AML.

### AML Vaccines

Vaccination is an attractive strategy for patients who are not eligible for HSCT or who relapse following HSCT. To date, three main types of vaccines are being tested in humans for AML: peptide, granulocyte macrophage colony stimulating factor (GM-CSF), and dendritic cell (DC) vaccines. Peptide vaccines involve the use of AML-associated antigens to stimulate a cytotoxic T cell response. Two common targets are Wilms tumor 1 (WT1) and PR1 (peptide derived from proteinase 3), both of which are overexpressed in AML. Recent results from a phase II clinical trial investigating the multivalent WT1 peptide vaccine galinpepimut-S (NCT01266083) suggest that the peptide vaccine is tolerated and may contribute to increase in overall survival (75). On the other hand, a PR1 peptide vaccine has been shown to induce antigen-specific immune responses in patients based on a Phase I/II trial (NCT00004918) (76,77). However, it is unclear when and how the vaccine should be used to produce optimal treatment efficacy (77). Another vaccination strategy is the use of gene-transduced tumor cell vaccine (GAVX) where cells are modified to express GM-CSF to recruit DCs to the site of intradermal vaccination to elicit an anti-tumour immune response (78). A phase II trial (NCT01773395) is being conducted to evaluate the efficacy of GVAX following allogeneic HSCT. Dendritic cell vaccination, which has the potential of presenting multiple tumour-derived antigens, is of particular interest for the treatment of AML due to the poor immunogenic nature of the disease (57,79). DCP-001 (DCPrime), a vaccine developed through the differentiation of the AML cell line DCOne into mature DCs, is currently recruiting for a phase II clinical trial (ADVANCE-II; NCT03697707) after demonstrating both safety and induction of immune responses in elderly AML patients in its phase I study (NCT01373515) (80). This strategy was designed to replace conventional DC vaccination approaches which involve administration of mature DCs that were isolated from the patients and matured *ex vivo*: an approach that is known to be costly, cumbersome, and logistically complex (28). Another DC-based treatment being tested in humans involves the vaccination with a hybridoma consisting of patient-derived AML cells fused with autologous DCs (81). In that study, 12/17 patients who had achieved remission experienced no recurrence after 57 months and showed expansion of disease-specific T cells which may help protect against relapse (81). This vaccination approach is being evaluated in combination with PD-1 blockade in a phase II clinical trial (NCT01096602).

### Chimeric Antigen Receptor T (CAR-T) Cell Therapy

CAR-T therapy has been greatly successful in the treatment of some hematologic malignancies but this accomplishment has yet to transfer to AML. Briefly, CAR-T cells are patient-derived T cells that have been genetically modified to recognize antigens expressed on the cancer cell’s surface (82). The development of CAR-T therapy for AML, however, has been much more challenging compared to that for B cell malignancies due to poor immunogenicity stemming from low mutation burden, the lack of AML-specific antigens leading to the risk of generating on-target off-leukemia toxicities, and the heterogeneous biology of the disease arising from various myeloid progenitors (57,62,82,83). Numerous targets are being explored in the preclinical and/or clinical development of CAR-T treatments for AML including CD33, CD123, FLT3(CD135), CD7, NKG2D, CD133, FRβ, LeY, and CLL-1 (82,84–91). While CAR-T cells are attractive due to their demonstrated success in other hematologic malignancies, CAR Natural Killer (NK) cells are also being investigated for AML as they are believed to be associated with lower risks of generating severe clinical toxicities such as cytokine release syndrome (92).

### Immune Checkpoint Inhibitors (ICPIs)

ICPIs have been associated with remarkable treatment outcomes in various solid tumours including NSCLC and melanoma (57). ICPIs involve the removal of immunosuppressive signals that are often used as a mechanism by cancer cells to evade immune detection. Although no ICPI has been granted approval for the treatment of AML, over 30 clinical trials are underway to evaluate the efficacy of clinically approved ICPIs as single agents or in combinations for newly diagnosed patients as well as post-remission and r/r AML patients. The role of ICPIs in AML will become clearer when results become available from these clinical trials.

### Immunomodulators for Immunogenic Cell Death Induction

Immunogenic cell death (ICD) has emerged in recent years as a popular immunotherapeutic concept that is being investigated pre-clinically and in early clinical trials. ICD is a form of cell death wherein cancer cells, in the process of treatment-induced death, emit certain molecular signals in a specific spatiotemporal pattern that result in the recruitment of immune cells, presentation of tumour-specific antigens, and activation of an adaptive immune response that consequently leads to tumour eradication and generation of immunological memory against future re-challenges (93–95). This “therapeutic vaccination” approach has garnered much attention as it has been shown that several existing chemotherapeutics are capable of inducing ICD, characterized by cell surface expression of calreticulin (CRT) and secretion of ATP and HMGB1 (93,96,97). Traditionally, chemotherapeutics are tested in immune-compromised pre-clinical models to ensure that the agents exert cytotoxic and not immunogenic effects. It is now known that certain chemotherapeutics (e.g. doxorubicin, oxaliplatin, mitoxantrone) induce ICD as a secondary mechanism, providing long-term immune protection in immune competent syngeneic models of cancer (95,96,98–100). While ample evidence suggest that ICD induction may provide cures and vaccination in pre-clinical models, evidence of ICD in humans are still being explored. Currently, over 20 clinical trials are investigating the immunological profile of existing chemotherapeutic regimens in various types of cancer (99). There are also multiple phase I/II clinical trials exploring the potential of novel immunomodulators to improve outcomes of standard chemotherapy or radiotherapy through ICD induction (NCT02906800, NCT02721056, NCT02805894). In a recent report, CRT exposure on leukemic blast cells has been correlated with increased AML-specific immune response, superior relapse-free survival and overall survival, suggesting that ICD-associated markers may be clinically relevant in the AML setting (101). While ICD has not been widely considered as an AML treatment strategy, it is important to note that anthracyclines such as doxorubicin and daunorubicin are used as SOC chemotherapy for AML patients while mitoxantrone, also a known bona fide ICD inducer, is an approved chemotherapeutic for r/r AML patients. Currently, there are over 40 trials involving the use of mitoxantrone to treat AML, suggesting that there are great interests in determining how to best use this potent chemotherapeutic. Use of in a combination setting where the anticancer immune response of mitoxantrone, other bona fide ICD inducers or immune-based treatments can be boosted using an adjuvant may prove to be an effective therapeutic/vaccination strategy (96).

## FLAM – Opening New Doors in AML

One of the combinatorial therapeutic strategies that has been employed in the treatment of AML is timed sequential therapy (TST). TST was established based on strategic sequential administration of cell cycle dependent anti-leukemic agents to augment the cytotoxic effect on leukemic cells (102,103). Over the years, TST has shown to yield favourable outcomes in AML patients, producing durable disease-free survival and improved remission rates (104–106). A TST that has shown great therapeutic potential is FLAM. FLAM is the sequential administration of flavopiridol followed by cytarabine and mitoxantrone (107). Flavopiridol is an anticancer agent derived from the plant alkaloid rohitukine that has shown to be effective against various types of cancer such as leukemia, prostate carcinoma, breast carcinoma, and bladder cancer (108–112). Flavopiridol works as a multi-cyclin-dependent kinase (CDK) inhibitor (CDK1, 2, 3, 4, and 9), effectively arresting cell cycle in the G1-S and G2-M phases (108,109,113–116). In AML, *in vitro* studies showed that flavopiridol induces synchronous cell cycling, increasing the proportion of cells in the S phase 48 h after flavopiridol exposure (102). This observation provided the basis for using flavopiridol in the FLAM regimen with S phase-specific agents like cytarabine and mitoxantrone. When administered as a component of FLAM, the combination regimen demonstrated, in a randomized multicentre Phase 2 trial, complete remission rates of nearly 70% in newly diagnosed poor-risk AML patients. This was nearly a 25% improvement in the complete remission rates compared to the 7 + 3 SOC regimen (103).

In addition to acting as a pan-CDK inhibitor, flavopiridol has also been shown to induce apoptosis through the downregulation of anti-apoptotic proteins such as Bcl-2 and Mcl-1 (117,118). As previously discussed, overexpression of Mcl-1 in AML is often synonymous with disease relapses and the ability of flavopiridol to repress the expression of Mcl-1 is believed to contribute to the synergism found in the FLAM regimen by potentiating the activities of cytarabine and mitoxantrone (119). Despite the remarkable improvement in complete remission rates following FLAM treatment, there were no difference in overall survival or event-free survival when compared to the conventional “7 + 3” regimen (103). Therefore, FLAM clearly has room for improvement.

One approach that our laboratory is considering is to enhance therapeutic effects of sequential flavopiridol, cytarabine and mitoxantrone is through reformulation using nanocarriers, such as liposomes. Nanomedicines are known to alter the pharmacokinetics of drugs, resulting in improved efficacy and reduced toxicities which ultimately lead to better treatment outcomes (120,121). To address the genetic heterogeneity of AML, broad-spectrum chemotherapy drugs have been used in the past and will continue to be used in the future. However, these drugs have associated side effects such as the anthracycline-induced cardiotoxicity that potentially life-threatening to AML patients (122). Additionally, commonly used AML drugs like cytarabine have been shown to have poor retention in blood and show enhanced antitumour activity when the circulation half-life of the drug is extended (123). The use of drug delivery systems, like liposomes, has proven capabilities in altering toxicity and extending circulation lifetime, which are features that are particularly relevant when considering treatments for patients with AML.

Vyxeos® represents one of the most recent success stories for nanomedicines in general and for their use in the treatment of AML in particular. As discussed, Vyxeos® delivers cytarabine and daunorubicin in liposomes at a fixed synergistic ratio and this demonstrates the ability of liposomes to control ratiometric dosing of anticancer drug combinations (124,125). Ratio-dependent dosing was based on *in vitro* data that showed that some drug combinations, but not all, work optimally at a specified drug-to-drug molar ratio. Prior to the inception of Vyxeos®, Mayer *et al*. liposomal formulations of several drug combinations based on the *in vitro* results showing that drug-drug ratio mattered in achieving synergy (126). For example, CPX-1 demonstrated the importance of ratiometric dosing to synergy maintenance between irinotecan and floxuridine (125). At high irinotecan/floxuridine ratios (10:1), irinotecan may antagonize the activity of floxuridine activity by causing cell cycle arrest in S phase (125,127). To build on this concept of ratiometric dosing, Mayer *et al*. experimented with various molar ratios of cytarabine- to-daunorubicin (1:10, 1:5, 1:1, 5:1, and 10:1) and found that only 1:1, 5:1, and 10:1 were synergistic, with the rest being either additive or antagonistic (125). Due to a lack of mechanistic explanations for the interaction, it can only be postulated that given cytarabine’s effects on newly synthesized DNA (therefore S-phase specific), it is possible that at some ratios, daunorubicin may antagonize cytarabine activity by arresting cells in G1 phase (128).

With Vyxeos® paving the way for nanomedicine and drug delivery systems, studies are underway to evaluate the use of various types of nanocarriers as means to improve the overall efficacy of anti-leukemic agents and to decrease the toxicities presented by these agents of interest. Of the various drug delivery platforms investigated, liposomes remain one of the most widely utilized systems (121,129).

In a study published in 2014, Tan *et al*. developed a method to co-encapsulate safingol and C2-ceramide, which are both bioactive sphingolipids that have displayed efficacy against AML but are limited clinically due to hemolytic toxicities (130). The resulting formulation significantly reduced the toxicities presented by the sphingolipids when compared to the free drug and the co-encapsulated formulation extended median survival time from 24 to 37 days compared to the singly encapsulated C2-ceramide (130). Other liposomal formulations such as liposomal daunorubicin-emetine (a protein synthesis inhibitor) and liposomal GTI-2040 (ribonucleotide reductase-targeting inhibitor) were also developed and examined preclinically against AML cells (131,132). Co-encapsulation of daunorubicin and emetine resulted in enhanced activity against MOLM-13 cells *in vitro*, with an increase in the induction of apoptosis by a factor of six (131). Similarly, liposomal GTI-2040 demonstrated significantly improved anti-tumour activity (decreased tumour size and prolonged overall survival of mice) when compared to the free drug (132). In addition to liposomal formulations, other polymer-based nanoparticle delivery systems have been explored for AML indications, including polymeric nanoparticles. In 2012, Simon *et al*. encapsulated ATRA (all-trans retinoic acid), a chemotherapy drug used to treat acute promyelocytic leukemia (an AML subtype), in polymeric nanoparticles made with poly-D,L-lactadie-co-glycolide (PLGA) (133). This polymer-based formulation allowed ATRA, an otherwise orally administered drug, to be available for intravenous injections, which can be beneficial for patients who are unable to swallow (133). Varshosaz *et al*. synthesized folate and retinoic acid grafted/dextran (FA-RA/DEX) polymeric micelles for targeted delivery of doxorubicin in AML (134). The doxorubicin-loaded micelles showed enhanced *in vitro* cytotoxicity against KG-1 cells when compared to the free drug (134). Another example of a nanocarrier demonstrating therapeutic improvements in AML includes dendrimer-based formulations. Dendrimers are nano-scale polymers that are globular in shape with branch-like configurations (135). Szulc *et al*. used dendrimers to formulate cytarabine triphosphate and the resulting cytarabine-complexed dendrimers significantly enhanced the cytotoxicity of cytarabine against 1301 cells (a T cell leukemia cell line) (135).

Therefore, in light of the recent success of Vyxeos® and the promising potential that liposomes have in improving AML therapeutics, we are developing a novel liposomal combination product derived from FLAM called “EnFlaM”: a product consisting of encapsulated forms of flavopiridol and mitoxantrone that will potentially amplify the synergy that exists between the two cytotoxic agents. In consideration of this effort, the advances in the development of nanoparticulate formulations of mitoxantrone and flavopiridol as single agents is summarized below. These nanoformulations are also summarized in Table II.

**Table II:** Nanoparticulate Formulations for Mitoxantrone in Preclinical and Clinical Investigation

**Formulation**	**Composition**	**Indication(s)**	**Development Stage**	**Ref.**
Liposome complexed mitoxantrone (LCM)	Soy phosphatidyl choline, cholesterol, phosphatidic acid, D,L-α-tocopherol	Breast cancer	I/II	(136,137)
∆pH Mitoxantrone liposomes	Soy phosphatidyl choline, cholesterol, DPPE-PEG_2000_	Leukemia	Preclinical	(138)
DSPC/Chol liposomes	DSPC, cholesterol	Leukemia	Preclinical	(139)
DMPC/Chol liposomes	DMPC, cholesterol	Leukemia, squamous cell carcinoma, colorectal cancer	Preclinical	(140,141)
Liposome-entrapped mitoxantrone Easy-To-Use (LEM-ETU)	DOPC, cholesterol, cardiolipin, alphatocopheryl acid succinate	Various cancers	I	(142,143)
Pegylated liposomal mitoxantrone 60 nm (PLM-60)	HSPC, cholesterol, DSPE-PEG_2000_	Leukemia, prostate cancer, non-Hodgkin’s lymphoma, various solid tumours, peripheral T cell lymphoma	I/II	(144–146)
Mitoxantrone polybutyl cyanacrylate (PBCA) nanoparticles	Butyl cyanacrylate, dextran-70, poloxamer 188	Leukemia, melanoma	Preclinical	(147,148)
Mitoxantrone polybutyl cyanacrylate nanoparticles (DHAQ-PBCA-NP)	Butyl cyanacrylate, dextran-70, sodium dithionite, sodium chloride	Hepatocellular carcinoma	II	(149–151)
Mitoxantrone solid lipid nanoparticles (MTO-SLN)	Lecithin, Compritol-888, surfactant S-40	Breast cancer	Preclinical	(152)
Mitoxantrone bovine serum albumin nanoparticles (MTO-BSANP)	Bovine serum albumin, glutaraldehyde, folic acid	Ovarian cancer	Preclinical	(153)
Mitroxantrone iron oxide magnetic nanoparticles	Iron oxide, dextran	Rhabdomyosarcoma	Preclinical	(154)

## Nanoparticulate Formulations of Mitoxantrone

Mitoxantrone is an active chemotherapeutic drug approved for the treatment of several cancer indications with primary side effects being cardiotoxicity, neutropenia, and bone marrow suppression (155). The controlled release of drugs from nanoparticles can help increase the circulation half-life, reduce renal clearance, and decrease the exposure of normal tissue to high drug concentrations (156). The resulting modified pharmacokinetics and biodistribution could improve the therapeutic index of the drug. Of the various nanoparticle formulations of mitoxantrone that were examined, liposomal mitoxantrone formulations remain the most extensively investigated for use in humans.

The first liposomal formulation of mitoxantrone was produced in the 1990s by Schwendener *et al*. and was based on the formation of complexes as a result of electrostatic interactions between the cationic drug and phosphatidic acid. The drug was encapsulated in liposomes comprised of soy phosphatidyl choline, D,L-α-tocopherol and cholesterol (Chol). The resulting mitoxantrone formulation was generally more effective and less toxic than the free drug in various tumour models including the murine lymphocytic leukemia model L1210 (136). The liposomal formulation was further evaluated in patients with advanced breast cancer in a Phase I/II study, where it was well tolerated and showed moderate antitumour activity (137). Despite the promising results, mitoxantrone was cleared from the blood circulation relatively rapidly and this triggered follow-up studies to improve the pharmacokinetics of the formulation. In one of the studies, mitoxantrone was encapsulated in novel soy phosphatidyl choline/ cholesterol liposomes modified with 1,2-dipalmitoyl-*sn*-glycero-3-phosphoethanolamine-*N*-[methoxy(polyethylene glycol)-2000] (DPPE-PEG_2000_) through a pH-gradient-mediated process. This resulting formulation was evaluated only in the preclinical setting and showed superior pharmacokinetic properties with a 40-fold increase of the area under the curve (AUC) (138).

To further optimize the drug release of liposomal mitoxantrone, additional studies were conducted by Madden *et al*. using the conventional 1,2-distearoyl-*sn*-glycero-3-phosphocholine (DSPC)/ Chol liposomes and sterically stabilized DSPC/Chol/ DPPE-PEG_2000_ liposomes. Mitoxantrone loading was mediated by a transmembrane pH-gradient and the resulting liposomes extended the survival time of L1210 bearing mice (139). The therapeutic activity of liposomal mitoxantrone was further influenced by the rate of release of the drug from the liposomes. When encapsulated in 1,2-dimyristoyl-*sn*-glycero-3-phosphocholine (DMPC)/Chol liposomes, mitoxantrone was released at a rate of 1.7 μg/μg lipid/h, which was significantly faster than when encapsulated in DSPC/Chol liposomes (<0.0257 μg/μg lipid/h) (140). The increased release rate resulted in significantly improved long-term survival of the animals treated with DMPC/Chol liposomal mitoxantrone (140). Similar observations were made in mice bearing A431 human squamous cell carcinoma and LS180 human colon cell carcinoma xenografts, where DMPC/ cholesterol liposomes resulted in greater delays in tumour growth compared with animals treated with other liposomal formulations (141).

In the 2000s, research on liposomal mitoxantrone continued with the use of 1,2-dioleoyl-*sn*-glycero-3-phosphocholine (DOPC)/Chol/cardiolipin liposomes (142). The encapsulation of mitoxantrone was based on the electrostatic interaction between the cationic drug and the negatively charged cardiolipin. The entrapment efficiency of the formulation was 99% and the mean vesicle size was 150 nm (142). The formulation was named liposome-entrapped mitoxantrone Easy-To-Use (LEM-ETU), it was only evaluated in a Phase I clinical trial by the company NeoPharm but no results were disclosed and the formulation has yet to progress further clinically (143).

To explore other liposomal formulations of mitoxantrone, Li *et al*. encapsulated mitoxantrone in PEGylated (polyethylene glycol-containing) liposomes using a transmembrane copper ion gradient and the formulation significantly enhanced the blood circulation time but did not improve the mean survival time of mice bearing L1210 leukemia cells (157). An additional study with PEGylated liposomes demonstrated that a formulation with hydrogenated soy phosphatidyl choline (HSPC)/ cholesterol/ 1,2-distearoyl-*sn*-glycero-3-phosphoethanolamine-*N*-[methoxy(polyethylene glycol)-2000] (DSPE-PEG_2000_) and high PEG density was the most active against S180 murine sarcoma (158). For this particular formulation, the effect of particle size on the therapeutic efficacy was investigated in a follow-up study (144). The results indicated that the small liposomes (60 nm) had the fastest release rate, exhibited less toxicity, and were the most efficacious (144). Continued research found that the level of antitumour activity of the small-sized formulation, termed PLM-60, directly correlates with increasing level of doses administered (145). The initial *in vivo* success of PLM-60 led to further investigation of the formulation in a Phase I study in patients with non-Hodgkin’s lymphoma and other malignancies (146). PLM-60 was found to be less toxic, potentially more efficacious, and longer circulating compared to unencapsulated mitoxantrone (146). Recently, in 2018, a randomized Phase I/II clinical trial (NCT03553914) has been scheduled to evaluate the toxicity and overall response rate of PLM-60 in patients with peripheral T cell lymphoma (PTCL).

In addition to liposomes, a polymeric nanoparticle formulation of mitoxantrone based on polybutyl cyanacrylate (PBCA) was tested as an anticancer agent in the clinics. Initial studies demonstrated that PBCA nanoparticles were not able to reduce the toxicity of mitoxantrone but were able to significantly reduce tumour volumes of mice bearing B16 melanoma (147,148). Due to their hepatic seeking tendencies, PBCA formulations serve as suitable drug delivery systems to target liver tumours, reducing toxicity to peripheral organs and enhancing the antitumour effects on hepatic malignancies (149). When a novel PBCA formulation, DHAQ-PBCA-NP, was evaluated in a murine model of orthotopically transplanted human hepatocellular carcinoma, the formulation resulted in inhibition of tumour growth and reduction of acute toxicity compared to free drug (150). This formulation was then evaluated in a Phase II clinical trial in patients with hepatocellular carcinoma. Intravenous administration of DHAQ-PBCA-NP resulted in increased cytotoxicity in hepatic tumours and improved median survival time by 2.23 months compared to patients treated with free mitoxantrone (151).

A myriad of other formulation methods were developed to encapsulate mitoxantrone. These include solid lipid nanoparticles (152) and micelles (159), which are examples of other lipid-based nanoparticles, and cationic surfactant cetyltrimethylammonium bromide (CTAB) micelles (160) and non-ionic surfactant micelles (161,162). Nanoparticles composed of other polymers such as poly(lactic acid-*co*-lysine) (PLA-PLL) (163), dextran (164), and hyaluronic acid/ chitosan were also used to deliver mitoxantrone (165). Another type of delivery system used were protein-based nanoparticles made with albumin (153) and beta-casein (166). Inorganic nanoparticles were also studied for the delivery of mitoxantrone and some examples include iron oxide magnetic nanoparticles (154,167), silica nanoparticles (168), and zeolite beta nanoparticles (169). The formulations listed here have only been tested *in vitro* or in preclinical models and none of these formulations have advanced further into clinical trials.

## Nanoparticulate Formulations for Flavopiridol

As discussed previously, flavopiridol is an active drug under investigation for many modalities of cancer including acute myeloid leukemia. However, clinical use of flavopiridol is largely hindered by its relatively low aqueous solubility (170) and high binding affinity to plasma proteins (171). In order to solubilize flavopiridol, it is dissolved in 30% hydroxypropyl beta-cyclodextrin/0.1 M citrate buffer (pH 4.52) to form a complex that can be clinically administered to patients (172). However, this formulation was reported to induce several side effects such as nausea/vomiting, diarrhea, fatigue, and neutropenia (173). Nanoparticles such as liposomes are well-documented to improve solubility and to provide steric barriers for encapsulated compounds (174–176). Therefore, the use of well-designed nanoparticle formulations can help increase the solubility of the drug, prevent the interaction with plasma proteins, and reduce toxicities associated with flavopiridol formulations.

Two different types of nanoparticles were used to encapsulate flavopiridol. In one study, flavopiridol was loaded by a transmembrane pH-gradient into liposomes composed of different lipids: HSPC/Chol, HSPC/Chol/ Tween-80, and HSPC/Chol/ DSPE-PEG_2000_ (177). HSPC/Chol/ DSPE-PEG_2000_, with a mean diameter of 120.7 nm and entrapment efficiency of 70.4%, was selected to conduct further pharmacokinetic studies in mice. Liposomal flavopiridol increased the elimination phase half-life from 57.0 min for free drug to 339.7 min for liposomal drug, decreased clearance from 0.036 L/min to 0.012 L/min, and augmented AUC from 3.4 min μmol/L to 10.8 min μmol/L (177). These results highlighted the capacity of liposomes to modulate the pharmacokinetic profile of flavopiridol.

In another study, flavopiridol was encapsulated into poly(lactic-*co*-glycolic acid) (PLGA) nanoparticles to produce a formulation for local delivery and sustained release of the drug (178). Drug release assays *in vitro* showed that the nanoparticles had a sustained release of flavopiridol for up to 3 days. More specifically, the formulation was prepared to inhibit astrocyte growth and inflammatory factor synthesis during the reparation of spinal cord injuries (178). In this context, flavopiridol nanoparticles decreased cell-cycle activation, reduced glial scarring, and facilitated survival and regeneration of neurons *in vivo*. Presumably, the use of PLGA nanoparticles of flavopiridol could be extrapolated to other diseases such as cancer, where modified pharmacokinetics resulting in extended sustained release of flavopiridol would be of advantage.

To our knowledge, the development of flavopiridol nanoparticles is limited to the two formulations described above. However, data from other flavonoids with similar structure, such as quercetin, suggest that flavopiridol could be loaded into a variety of different nanomaterials. There are some examples of quercetin nanoparticles designed for the treatment of cancer. Quercetin was entrapped in HSPC/Chol/ PE-PEG_2000_ liposomes (179), soy lecithin/ cholesterol/ PEG_4000_ liposomes (180), egg sphingomyelin/ cholesterol/ ceramide-PEG_2000_ liposomes (181), and lipoid S75/ oleic acid liposomes (182). Additionally, polymers like poly(lactic acid) nanoparticles (183), superparamagnetic iron oxide nanoparticles (184), and mesoporous silica nanoparticles (185) have all been shown to encapsulate quercetin. Additionally, a quercetin-encapsulated liposomal product was successfully developed in our lab using a metal complexation technology that enhanced quercetin’s circulation longevity and improved its apparent solubility (186). Thus, it would be of particular interest to see if these formulations can be used to encapsulate flavopiridol to increase the therapeutic potential of the drug.

## EnFlaM - a Simpler Method to Achieve Time Sequenced Treatments like FLAM?

The intriguing improvements in complete remission rates achieved by the FLAM regimen has shed new light on timed sequential therapy (TST) in the treatment of AML. However, FLAM is a treatment with limitations that could be possibly overcome by altering the pharmacokinetics and biodistribution of the drugs. As proposed, EnFlaM will be a liposomal combination product of mitoxantrone and flavopiridol that could potentially be a new treatment regimen implemented to address the challenges of AML treatment. We believe that EnFlaM will be a better alternative to FLAM with the potential of eliminating the need for TST. EnFlaM could be designed to achieve optimized drug delivery and enhanced therapeutic activity.

The principle of TSTs is to expose cancer cells to cell cycle-dependent drugs in sequence in an effort to achieve synergistic effects. As discussed previously, nanoparticles like liposomes are drug delivery systems whose lipid composition and structure can be varied to achieve desired release kinetics ranging from rapid release to intermediate release to slow release, providing long-term sustained drug delivery – an effect that is separate and distinct from the ability of nanoformulations to deliver contents to selected cell populations through nanoparticle cell interactions (187). The variety of lipid compositions and loading methods provide great versatility in the design of suitable formulations that can be selected for development (187). Liposomes have previously been shown to increase the plasma concentration of drugs, mediating an increase in the drug’s anticancer activity (188). Due to its high tendency to bind serum protein and its rapid elimination from the plasma, flavopiridol is particularly well-suited to be therapeutically enhanced through liposomal delivery (189). Therefore, when encapsulated, flavopiridol in an appropriate liposomal formulation should be therapeutically more active. Similarly, the therapeutic potential of mitoxantrone benefits from liposomal encapsulation. Mitoxantrone, although developed as a less cardiotoxic agent compared to anthracyclines, also causes dose-limiting cardiotoxicity and the use of liposomes can curb this cardiotoxicity (190,191). Therefore, an encapsulated formulation of mitoxantrone should be less toxic and better tolerated. Preliminary *in vitro* studies have shown that flavopiridol and mitoxantrone interact synergistically in a ratio-independent way (unpublished data) and it is reasonable to consider these data in the development of more efficacious and less toxic formulations. We aim to capture the synergistic potential of flavopiridol and mitoxantrone through liposomal technology, but recognize that there is also a potential to capture synergistic toxicity when using the combination product.

Additionally, one of the causes of AML relapses is attributed to leukemic stem cells and these stem cells home to and engraft within bone marrow, where they remain quiescent and protected from the effects of cell cycle-dependent chemotherapeutic agents (192–194). With the use of liposomes or other nanotechnology-based formulations, it will be possible to improve delivery of cytotoxic agents to the bone marrow to target leukemic stem cells. For example, liposomes have previously been shown to be preferentially taken up by bone marrow macrophages and this process facilitates the transport of drugs along with the liposomes to the bone marrow (195,196).

## Conclusions and Future Perspectives on AML

xThe standard of care for AML has remained almost unchanged for more than 40 years. Only recently in 2017 and 2018, a series of new treatments that provide therapeutic benefits especially to patients of older age or those who have recurrent disease have been approved for clinical use. The emergence of these treatments, namely midostaurin, enasidenib, Vyxeos®, gemtuzumab ozogamicin, venetoclax, and glasdegib, provide new hopes for patients in the r/r setting. With the development of novel immunotherapeutics, a plethora of clinical trials are being conducted to improve the prognosis of AML patients and to overcome the inherent challenge of activating an anticancer immune response against a heterogeneous disease that is poorly immunogenic.

However, AML still presents a number of clinical challenges and novel therapeutic strategies are necessary to provide additional treatment options for patients with high-risk disease. The FLAM regimen based on timed sequential therapy with flavopiridol, mitoxantrone and cytarabine has shown favourable outcomes with remarkable improvements in complete remission rates in AML. However, the FLAM regimen has some limitations and failed to improve overall survival. Thus, we proposed the use of nanomedicine to circumvent these limitations by introducing EnFlaM. “EnFlaM” will consist of a liposomal combination product of mitoxantrone and flavopiridol that will potentially improve the pharmacokinetics and biodistribution and increase the therapeutic efficacy of the drugs.

Through the lessons we learned from reviewing the clinical challenges of AML and the breakthroughs with recently approved treatments, we expect that the most effective treatments, as in the current standard of care of AML and other cancers, would rely on multi-modal therapeutics comprising of combinations of chemotherapy, targeted therapy, or immunotherapeutic modalities. It is therefore anticipated that many more clinical trials will be designed to focus on combining two or more drugs with immunotherapeutic agents. For instance, the use of a PD-1-targeting checkpoint inhibitor in conjunction of the DC/AML fusion vaccine is being investigated in a phase II clinical trial for AML patients who have achieved remission after chemotherapy (NCT01096602). There are also clinical trials combining various immunotherapeutic strategies with standard treatments (e.g. NCT03286114, NCT00006045) and studies where combinations of different ICPIs are being explored (e.g. NCT01822509, NCT02397720). With novel nanoformulations such as Vyxeos® becoming standard treatment for subpopulations of AML patients and the potential of nanoparticles to enhance immunotherapeutic effects, it will be of great interests to evaluate treatment combinations of EnFlaM with immune checkpoint inhibitors approved for other cancer indications to determine whether or not improved immunotherapeutic effects and extended survival can be achieved.
